# Supplementary figures and images for: Mouse Navigation Strategies for Odor Source Localization
Source: Front Neurosci. 2020 Mar 20;14:218. doi: 10.3389/fnins.2020.00218 (PMC7101161; doi:10.3389/fnins.2020.00218)

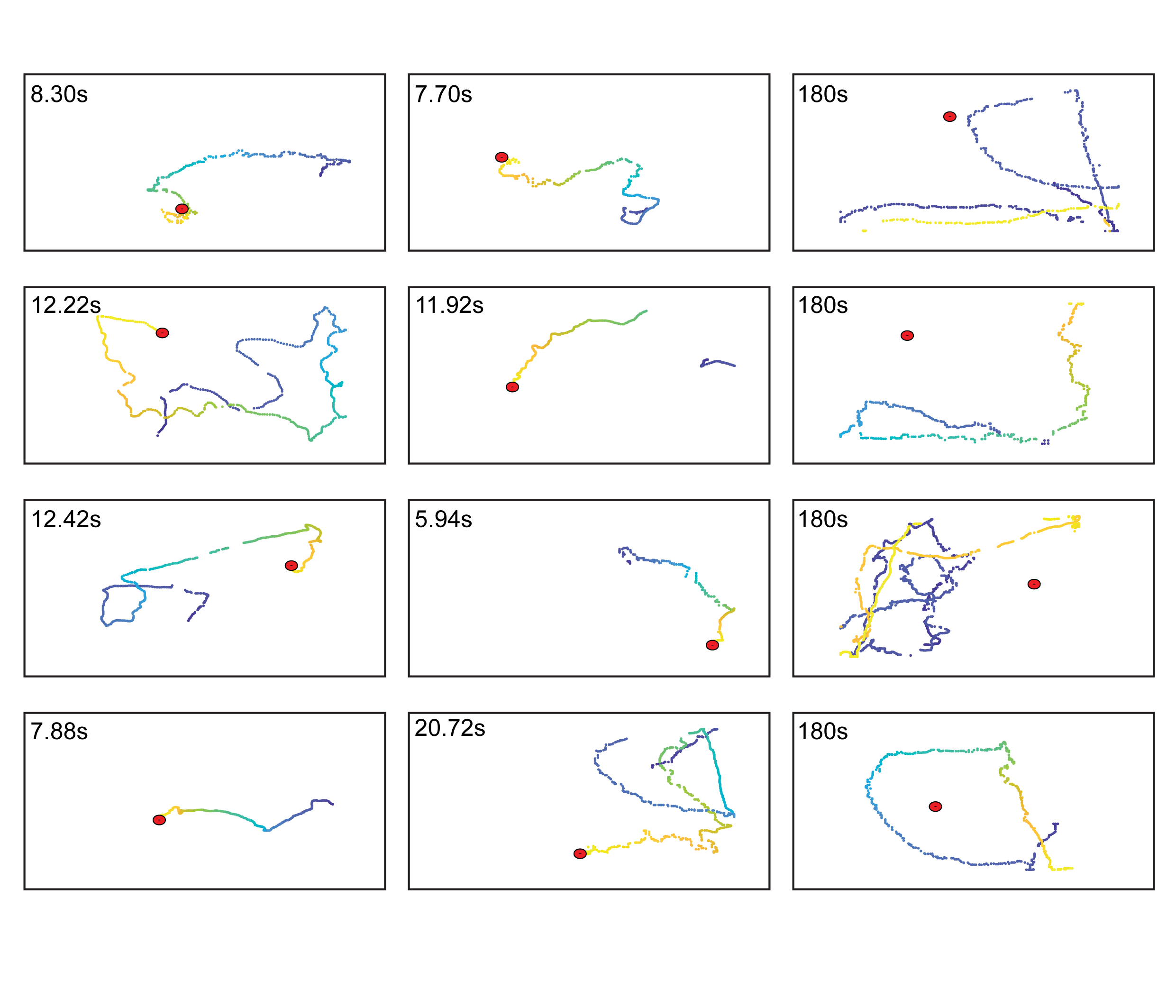

Supplement: FIGURE S1 — Example traces. Successful trials (left and center panels) have the time to spot indicated by the text. The relative time of each position sample is indicated by the color. Unsuccessful trials (right panel) have the total trial time indicated by the text. Color represents the relative time of each position sample. Spot positions are indicated by red circles. [file Image_1.tif]

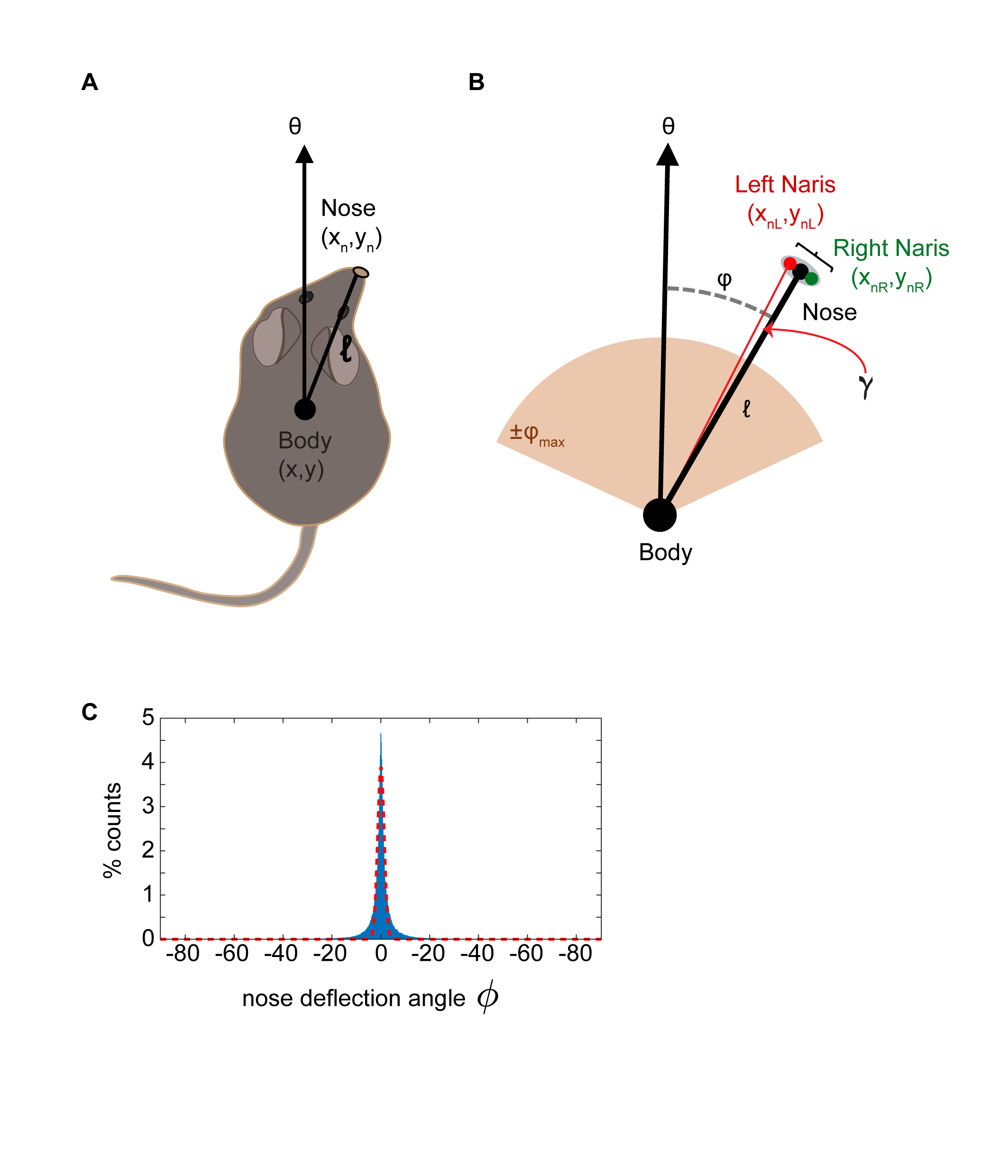

Supplement: FIGURE S2 — Detailed model geometry. (A) Model specification of the body position (x, y), and the body angle θ superimposed on a diagram of the mouse. The nose position (xn, yn) is the mean position between the left and right nares. (B) Nose angle ϕ (gray dashed line) is the angle between θ and nose position, and is constrained by a maximum angle ϕmax. The head-body length l is the distance between the body position and the nose position, which is sub-divided into two nostrils separated by a distance dnares. The angle from the center of the nose to the edge of the nostril is γ. (C) The distribution of mouse nose deflection anglesϕ (blue bars) is approximately Gaussian {dashed red line; a∗exp[−((x−b)/c)∧2]; a = 0.04, b = 0.06, c = 1.92}. [file Image_2.tif]

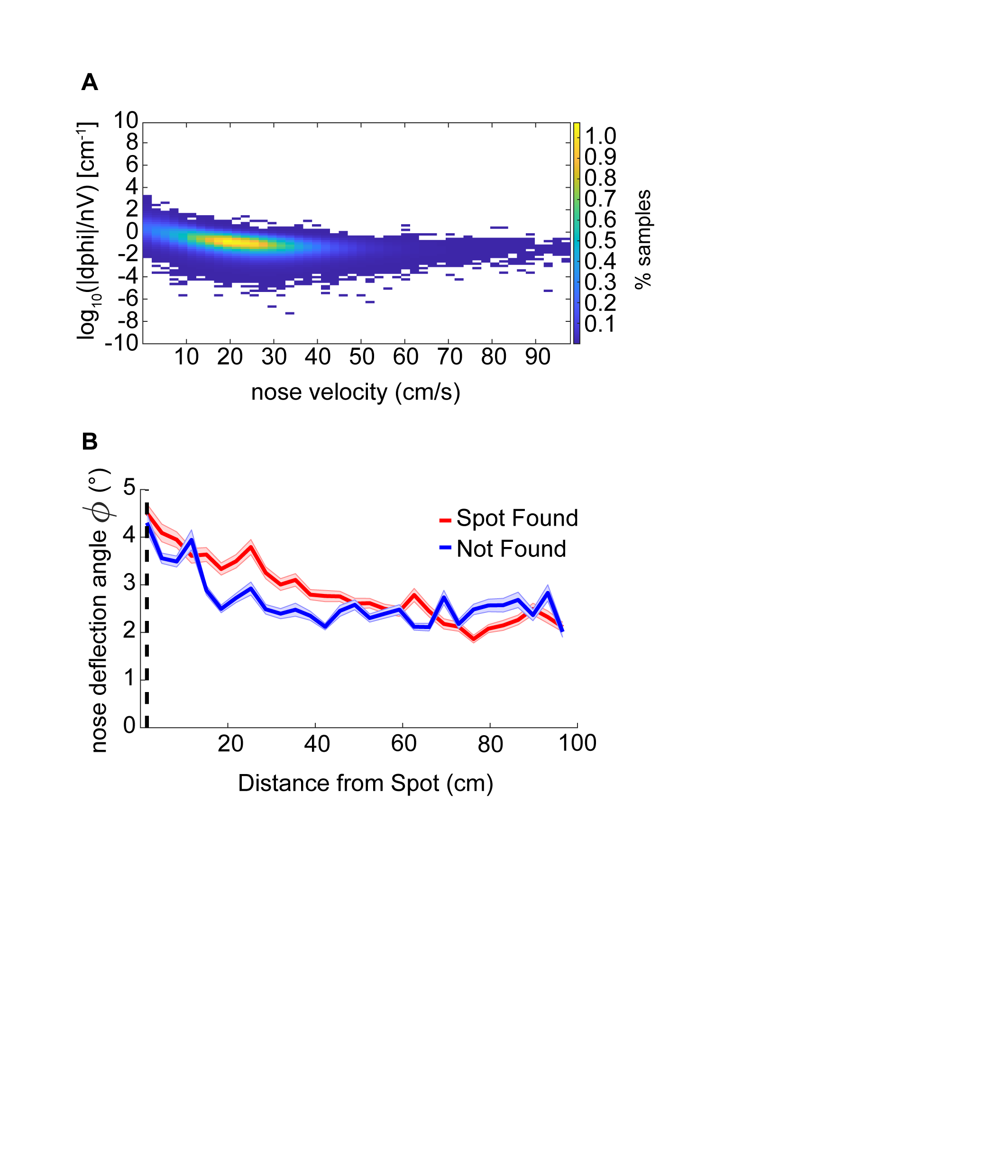

Supplement: FIGURE S3 — Additional casting analyses. (A) Curvature as measured by log10 (—dPhi—/nV) depends on nose velocity. The color map indicates the percentage of samples in each bin. (B) Averaged across successful trials, nose deflection angle ϕ increases as the distance to the spot decreases (line = mean ± SEM; ANOVA; p = 2.18E−56, F = 12.82, df = 28). [file Image_3.tif]

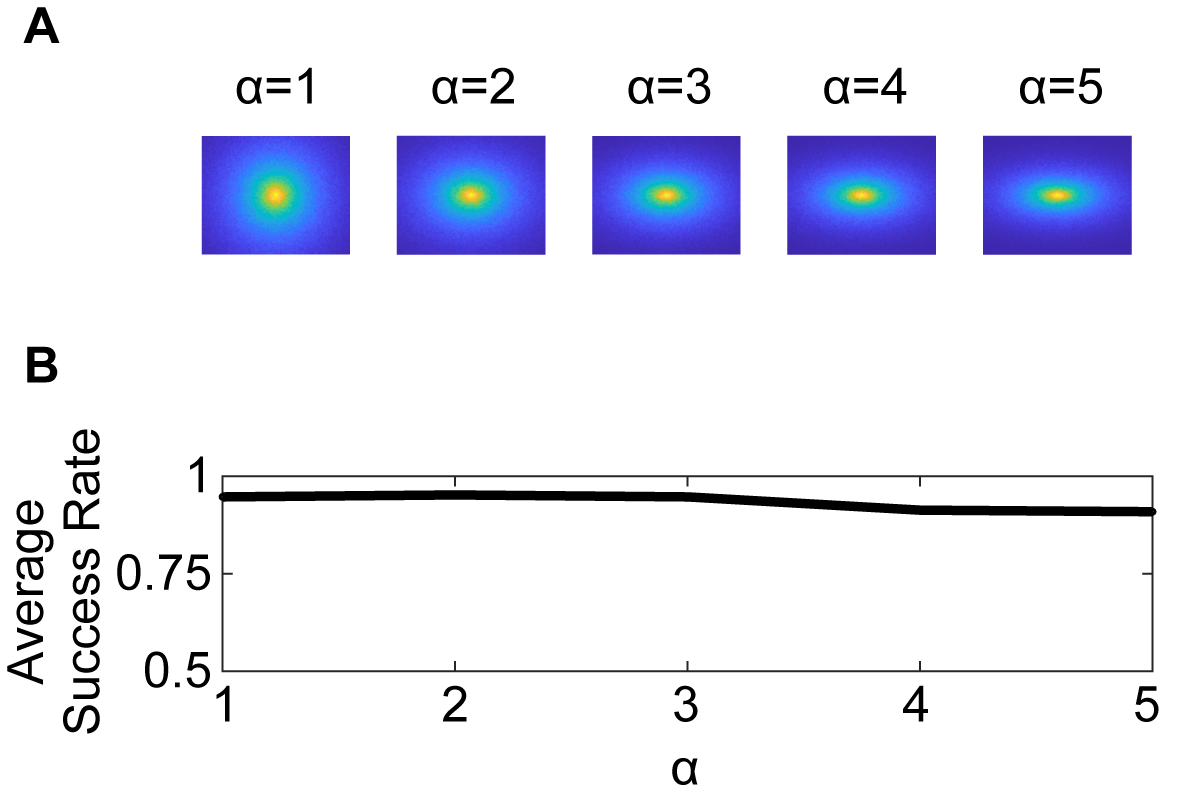

Supplement: FIGURE S4 — Non-symmetric plume analysis. (A) To test the effect of removing radial symmetry on model performance, we created elliptical sources using an additional shape parameter α. Increasing the shape parameter resulted in more longitudinally skewed plumes. (B) Performance was not strongly affected by removing the assumption of radial symmetry. [file Image_4.tif]
