# Supplementary material for: Mouse Navigation Strategies for Odor Source Localization
Source: Front Neurosci. 2020 Mar 20;14:218. doi: 10.3389/fnins.2020.00218 (PMC7101161; doi:10.3389/fnins.2020.00218)
Supplement: TABLE S1 — Logistic regression analysis on spot-finding success in mice. Significant variables in bold. [file Table_1.docx]

**SUPPLEMENTAL TABLE 1**

| **Variable** | **p value** | **β value** |
| --- | --- | --- |
| Experimenter | 4.20E-01 | -0.43 |
| **Baited** | **4.68E-12** | **2.51** |
| Concentration | 4.93E-03 | 0.56 |
| Order of Trial | 0.08 | 0.23 |
| Trial | 0.98 | 4.47E-03 |
| Mouse | 0.11 | **0.16** |
| **Initial Distance** | **6.54E-08** | **4.53E-02** |
| Initial Orientation | 0.79 | 3.26E-04 |
| **Mean Body Velocity** | **6.89E-06** | **-0.29** |
| **Mean Nose Velocity** | **1.07E-04** | **0.27** |
| **Displacement Ratio** | **4.19E-19** | **-0.38** |
| **Mean Curvature** | **2.56E-03** | **1.4** |
| **Exploration %** | **1.65E-15** | **15.51** |
| Mean Nose Acceleration | 0.01 | -0.05 |
| Time Stopped | 0.22 | -0.02 |
