# Supplementary material for: Mouse Navigation Strategies for Odor Source Localization
Source: Front Neurosci. 2020 Mar 20;14:218. doi: 10.3389/fnins.2020.00218 (PMC7101161; doi:10.3389/fnins.2020.00218)
Supplement: TABLE S2 — Logistic regression analysis of CSM parameters on spot-finding success. Significant parameters are in bold. [file Table_2.docx]

**SUPPLEMENTAL TABLE 2**

| **Variable** | **p value** | **β value** | **Range** |
| --- | --- | --- | --- |
| **d_nares_** | **1.00E-50** | **0.54** | **0.01 - 1cm** |
| k_c_ | 4.84E-03 | -0.1 | 0 - 1 |
| **k_binaral1_** | **6.86E-26** | **1.27E-03** | **0.01 - 300** |
| **k_binaral2_** | **7.33E-106** | **0.16** | **0 - 5** |
| **k_v_** | **2.63E-47** | **0.62** | **0 - 1** |
| **ℓ (neck length)** | **1.35E-191** | **0.26** | **0.01 - 10cm** |
| **Mean Curvature (dist < 30cm)** | **6.13E-09** | **0.6** | **Dependent Variable** |
| **Mean Nose Velocity (dist < 30cm)** | **2.30E-19** | **-0.016** | **Dependent Variable** |
| **n_c_** | **4.67E-09** | **-0.043** | **0 - 5** |
| **n_v_** | **4.76E-160** | **0.22** | **0 - 5** |
| **σ_max_** | **1.11E-39** | **0.77** | **0.01 - 1** |
| σ_min_ | 2.50E-01 | 0.074 | 0.01 - 1 |
| τ | 6.40E-01 | -0.02 | 0.01 - 1 |
| **V_max_** | **9.26E-54** | **0.03** | **20 - 40cm/s** |
